# Chemokines and cellular plasticity of ovarian cancer stem cells

**DOI:** 10.18632/oncoscience.181

**Published:** 2015-07-25

**Authors:** Weiping Zou, Max S. Wicha

**Affiliations:** Department of Surgery, Graduate Programs in Immunology and Tumor Biology, University of Michigan Comprehensive Cancer Center, University of Michigan School of Medicine, Ann Arbor, MI, USA; Departments of Medicine and Tumor Biology, University of Michigan Comprehensive Cancer Center, University of Michigan, Ann Arbor, MI, USA

**Keywords:** CCL5, chemokine, cancer stem cell, immunity, metastasis

The diversity of cancer is generated through genetic alterations as well as by epigenetic events regulated by the tumor microenvironment. At the apex of this hierarchy are cancer stem cells (CSCs) which are characterized by their ability to self-renew, as well as to generate the cells constituting the tumor bulk. CSCs contribute to cancer metastasis and therapeutic resistance [1]. It is now clear that cellular interactions within the tumor microenvironment regulate both the cancer stem cell and bulk cell components which play an important role in tumor progression and metastasis. Deconvoluting the role of various components in the human cancer microenvironment and elucidating the mechanism of interaction has proved to be a great challenge. Recent studies have demonstrated that different immune components including Th22 cells [2] and myeloid derived suppressor cells (MDSCs) [3] are involved in the control of cancer stemness. Chemokines are important players in the immune responses. For some time it has been known that chemokines are able to target and recruit specific immune subsets into the microenvironment. For example, CXCL12 [4] and CXCL20 [5] mediate tumor trafficking of plasmacytoid dendritc cells (4) and regulatory T cells [5], respectively. These immune cell subsets can mediate immunosuppression and consequently promote cancer progression. Interestingly, chemokines may directly target tumor cells and affect tumorigenesis. CXCL8 (IL-8) [6] has been demonstrated to be involved in cancer stem cell biology and cancer cell metastasis. Furthering this work, Zhu et al, in this issue of *Oncoscience*, describe a new role for cancer stem cell-derived CCL5 in ovarian cancer metastasis.

Ovarian cancer stem cells are enriched in cell fractions expressing CD133^+^ and ALDH-1^+^ [3, 7]. In an attempt to examine the role of CCL5 in cancer metastasis, Dr. Zhu and colleagues employed several complementary approaches to test the hypothesis that cancer stem cell-derived CCL5 played a role in endowing CSCs with invasive properties. They found that in the presence of CD133^+^ CSCs, CD133^−^ cancer cells undergo an epithelial-mesenchymal transition (EMT)-like process and display enhanced metastatic capacity in *in vitro assays* as well as *in vivo* xenografts. They demonstrated that CD133^+^, but not CD133^−^ cancer cells, expressed CCL5, with CCL5 signaling blockade abrogating and recombinant CCL5 mimicking these effects. In further support of the potential clinical significance of CCL5 and its receptors in tumorigenesis, they detected elevated expression of CCL5 and its receptors CCR1, CCR3 and CCR5 in human and murine metastatic epithelial ovarian carcinomas. To gain mechanistic insight into this process, they observed that paracrine CCL5 from CD133^+^ ovarian cancer stem cells activated the NF-κB signaling pathway in CD133^−^ cells *via* binding CCR1, CCR3 and CCR5, thereby inducing EMT and tumor invasion. Therefore, it is reasonable to conclude that at least in this experimental setting, CCL5 and/or CCL5 expressing CSCs promote ovarian cancer EMT and potential metastasis.

Although this manuscript represents a significant advance, many questions remain to be answered. The authors propose that CCL5 converts non-CSCs into CSCs through an EMT. However, they do not exclude the possibility that there are more than one form of CSCs in ovarian cancer since previous studies have suggested that CSCs can exist in alternative EMT- and MET-like states [8]. Thus, induction of EMT may represent transition in CSC state, rather than true induction of CSCs. In addition, the authors do not examine the role of other cell types in the tumor microenvironment which are capable of CCL5 expression. To what extent CD133^+^ cell-derived CCL5 is important in ovarian cancer metastasis in patients is not known. In addition, since these studies utilize established cell lines, it is unclear whether primary ovarian cells show similar cytokine expression patterns. Despite these limitations, this work adds to increasing evidence for the importance of CSCs and their regulation by cell-cell interactions in the tumor microenvironment. Furthermore, these studies suggest the possibility of inhibiting ovarian CSCs by blocking CCL5 signaling.
